# Transcriptional Network in *Colletotrichum gloeosporioides* Mutants Lacking Msb2 or Msb2 and Sho1

**DOI:** 10.3390/jof8020207

**Published:** 2022-02-21

**Authors:** Na Liu, Fanli Meng, Chengming Tian

**Affiliations:** The Key Laboratory for Silviculture and Conservation of Ministry of Education, College of Forestry, Beijing Forestry University, Beijing 100083, China; naliums@bjfu.edu.cn

**Keywords:** *Colletotrichum gloeosporioides*, membrane receptor, signal recognition and transduction, pathogenicity

## Abstract

*Colletotrichum gloeosporioides* is a hemibiotrophic ascomycetous fungus that causes anthracnose in many plants worldwide. During infections, *C. gloeosporioides* produces an appressorium in response to various plant surface signals. However, the mechanism mediating host surface signal recognition remains unclear. In this study, *C*. *gloeosporioides* *ΔCgMsb2* and *ΔCgMsb2Sho1* mutants lacking hypothetical sensors of plant surface signals were examined. The mutations in *ΔCgMsb2* and *ΔCgMsb2Sho1* adversely affected conidial size and sporulation, while also inhibiting growth. Significant transcriptional changes were detected for nearly 19% and 26% of the genes in *ΔCgMsb2* and *ΔCgMsb2Sho1*, respectively. The lack of these plasma membrane receptors altered the expression of specific genes, especially those encoding hydrolases, ABC transporters, and mitogen-activated protein kinases (MAPKs). The encoded MAPKs participate in the signal transduction of ERK and JNK signaling pathways, activate downstream signals, and contribute to metabolic regulation. Our data demonstrate that the *C*. *gloeosporioides* membrane proteins Msb2 and Sho1 affect gene regulation, thereby influencing conidial growth, metabolism, and development. These findings provide new insights into the regulation of *C*. *gloeosporioides*’s development and infection of plant hosts.

## 1. Introduction

*Colletotrichum* Corda is one of the top 10 fungal plant pathogens in molecular plant pathology [1]. *Colletotrichum gloeosporioides*, which is an important plant pathogen, is widely distributed in tropical, subtropical, and temperate regions, where it can infect and damage gramineous and woody plants [2,3,4]. More specifically, *C. gloeosporioides* is a hemibiotrophic fungus that produces conidia, which initiate infections at the leaf surface. First, after conidia are activated, their tips secrete mucilage that enables them to adhere to the leaf surface. Next, bud tubes form on the surface of the conidia and then quickly undergo polar growth and cell division. The polar growth then ceases at the bud tube end, which subsequently expands to form a special infection-related structure called an appressorium [5]. After reaching a certain turgor pressure, the mature appressorium penetrates the plant epidermis and invades the host tissue to produce primary hyphae [6]. The continuous expansion of *C. gloeosporioides* in plant cells results in the substantial production of secondary hyphae that secrete a large number of cell-wall-degrading enzymes, resulting in the degradation of plant tissues. Additionally, the hyphae obtain nutrients for their growth and development. Finally, the pathogen causes leaf necrosis (e.g., in poplar trees), which is a typical symptom of anthracnose [7]. The key of *C. gloeosporioides* infecting plants is the recognition on the plant surface and the formation of appressorium. 

On the plant surface, appressorium formation involves specific signals, including epidermal wax and keratinocytes [8], which provide information regarding the physical properties of the plant surface (e.g., hydrophobicity and hardness) [9]. These stimuli are perceived by fungal cell surface sensors, after which the signals are transmitted to cells via downstream signaling pathways. Meanwhile, appressorium formation is a complex and highly regulated process involving modifications to the cell wall [10,11], gene expression [12,13,14,15], and the cytoskeleton [16,17,18]. Thus, characterizing the molecular mechanisms underlying the perception and integration of these stimuli is important for elucidating how plant pathogens enter the host. Sensing the host surface signals is an important part of pathogen infections, with hypothetical sensors playing an indispensable role. In *Magnaporthe grisea*, Sho1 and Msb2 have overlapping functions in recognizing different plant surface signals, while also regulating the phosphorylation of Pmk1 [19]. In cells, mitogen-activated protein kinase (MAPK) is an important signal transducer and regulator of a variety of physiological processes [20]. The following four MAPK subfamilies have been identified in eukaryotic cells [21]: extracellular signal-regulated kinase (ERK) 1/2, p38MAPK, c-Jun, and ERK5. In *Ustilago maydis*, Sho1 and Msb2 are specifically involved in the perception of host surface hydrophobicity [22] and have overlapping functions related to appressorium development and plant infections. In many plant pathogens, homologs of the yeast protein Msb2 have been identified as putative sensors involved in plant surface signal recognition, pathogenicity, and Pmk1 (i.e., MAPK) activation [23]. We previously demonstrated that CgMsb2 plays a significant role in the recognition of various host surface signals. The lack of CgMsb2 leads to a defective appressorium formation and decreased pathogenicity. In addition, CgSho1 plays a secondary role and cooperates with CgMsb2 to jointly regulate host signal recognition as well as pathogenicity. However, the regulation of gene changes related to CgMsb2 and CgSho1 is unclear, and how to activate the downstream signal pathway remains to be explored. To clarify the host surface signal recognition and transduction mechanism of pathogens, we explored the regulation of CgMsb2 and CgSho1 through transcriptomes and screened the key regulatory genes and signal pathways in the early stage of appressorium formation.

In the present study, we performed a transcriptome analysis of *C. gloeosporioides* mutants lacking Msb2 (*ΔCgMsb2*) or lacking Msb2 and Sho1 (*ΔCgMsb2Sho1*). More specifically, their transcriptomes were sequenced and analyzed to reveal differences in gene expression profiles.

## 2. Materials and Methods

### 2.1. Fungal Strains and Culture Conditions

The WT *C. gloeosporioides* strain CFCC80308, which was used for analyzing transcriptome profiles, was isolated from *Populus* × *beijingensis* in Beijing, China. The *ΔCgMsb2* and *ΔCgMsb2Sho1* mutants were generated in one of our previous studies [24]. For all strains, conidial suspensions (in 50% glycerol) were kept at −80 °C for long-term storage. All strains were cultured at a room temperature of 25 °C in plates containing potato dextrose agar (PDA) medium (200 g of potato, 20 g of glucose, and 15 g of agar per liter).

### 2.2. Analyses of Vegetative Growth and Conidiation

Conidial suspensions of the WT and mutant strains were prepared using sterilized deionized water (10^6^ conidia/mL). A 1 µL aliquot of the conidial suspension of each strain was used to inoculate PDA medium in plates, which were then incubated at 25 °C for 5 days. The colony diameter was measured daily starting on day 2. After the 5-day incubation, the spores on the mycelium were washed with 2 mL sterile water and then the conidial yield and size were determined for each strain. Additionally, the WT and mutant spores were examined using an optical microscope and photographed. All experiments were performed three times.

### 2.3. RNA Extraction

The conidia on the mycelium were washed with sterile water and centrifuged. After removing the supernatant, the conidia were immediately frozen in liquid nitrogen. Total RNA was extracted using the TRIzol reagent (Invitrogen, Carlsbad, CA, USA). RNA degradation and contamination were assessed by 1% agarose gel electrophoresis, whereas RNA integrity was evaluated using the 2100 Bioanalyzer (Agilent, Mainz, Germany). The RNA concentration was determined using the NanoDrop 8000 spectrophotometer (NanoDrop, Waltham, MA, USA). High-quality RNA samples were retained for transcriptome sequencing.

### 2.4. Preparation of Transcriptome Sequencing Libraries

For each strain, the total RNA (3 μg) was used for constructing transcriptome sequencing libraries using the Illumina kit (San Diego, CA, USA). Briefly, the mRNA among the total RNA was enriched using oligo-(dT) magnetic beads and then immediately treated with a fragmentation buffer. The resulting mRNA fragments served as the template for synthesizing first-strand cDNA using random hexamers, after which buffer, dNTPs, RNase H, and DNA polymerase I were added to synthesize the second cDNA strand. The cDNA was subsequently purified using AMPure XP beads. Following an end-repair step, the cDNA was modified by the addition of a tail sequence and a sequencing adapter. A fragment size selection step was performed using AMPure XP beads, which was followed by a PCR amplification to complete the construction of the sequencing library.

### 2.5. Transcriptome Analysis and Sequence Assembly

The transcriptome analysis was performed using three biological replicates. The prepared libraries were sequenced on the Illumina HiSeq 2000 system at the Beijing Genome Institute, which resulted in 150 bp paired-end reads. Raw reads were filtered by eliminating low-quality reads using the Trimmomatic program (version 0.33) (RWTH Aachen University, Aachen, Germany). The retained reads were aligned to the reference transcriptome to identify new genes and quantify gene expression using HISAT2 software [25]. The aligned reads were assembled using StringTie [26] to construct complete transcripts and analyze expression. The reads were aligned to the reference genome according to a Burrows–Wheeler transformation-based method [27] to calculate the number of mapped reads. The fragments per kilobase million (FPKM) value was calculated for the fragments with two paired-end reads.

### 2.6. Analysis of Differential Gene Expression

Differential expression between samples was analyzed using DESeq2 [28]. The following criteria were used to identify significant DEGs: fold-change ≥ 1.5 and *p* < 0.01. The false discovery rate was calculated on the basis of the *p* value. Multiple public databases were screened to assemble the transcripts in the transcriptomes. Gene Ontology terms were used to functionally characterize gene products and clarify their cellular localization. The DEGs were classified into the three main GO categories (biological process, cellular component, and molecular function) using the GOseq R package [29]. Additionally, the R package was used to calculate adjusted *p* values. An adjusted *p* < 0.05 was used as the threshold for determining the significance of the annotation of a DEG with a particular GO term. The adjusted *p* value was adjusted according to the Benjamini and Hochberg method (false discovery rate < 0.05) [30] to identify significant DEGs. The KEGG database was used to further elucidate the functions of the identified DEGs in biological systems, including cells, organisms, and ecosystems. The hypergeometric test was used to analyze the significant enrichment of KEGG pathways among the DEGs.

### 2.7. qRT-PCR

The samples used for the RNA-seq analysis were also used for the qRT-PCR analysis, which was conducted to evaluate the reliability and reproducibility of the transcriptome data. Primer pairs for the selected genes (Supplementary Table S3) were designed using Primer Premier 6.0. First-strand cDNA was synthesized using the Hifair^®^ Ⅱ 1st Strand cDNA Synthesis SuperMix for qPCR (with gDNA digester plus) (Yeasen Biotechnology, Shanghai, China). The qRT-PCR analysis was performed using the 2 × HQ SYBR qPCR Mix (Zoman Biotechnology, Beijing, China). The CFX Connect Real-Time PCR instrument (Bio-Rad, Hercules, CA, USA) and CFX Manager Software 3.0 were used. Each sample was analyzed in triplicate and the average values were calculated. Relative gene expression levels were determined according to the 2^−∆∆Ct^ method [31].

### 2.8. Statistical Analysis

Raw data were analyzed using the SPSS 17.0 program (SPSS Inc., Chicago, IL, USA). In this study, significant differences between samples (*p* < 0.05 and *p* < 0.01) were determined by a one-way analysis of variance (Duncan’s test).

## 3. Results

### 3.1. ΔCgMsb2 and ΔCgMsb2Sho1 Exhibit Defects in Vegetative Growth and Conidiation

**Figure 1:**
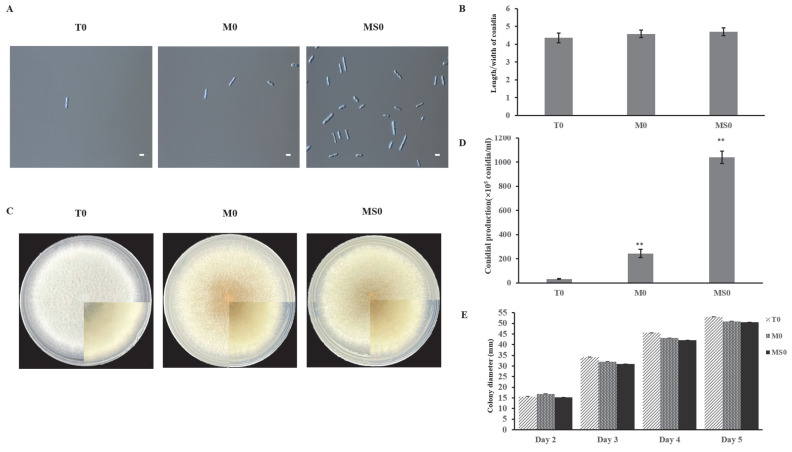
Effects of the lack of Msb2 and Sho1 on conidial size, growth, and sporulation. (**A**) Images of the conidia from WT, *ΔCgMsb2*, and *ΔCgMsb2Sho1* strains. Conidia were washed with 2 mL sterile water. Scale bar: 10 µm. (**B**) Length/width ratio of conidia. Error bars represent the standard deviation for three independent replicates, each comprising three technical replicates. Asterisks indicate significant differences (*p* < 0.05). (**C**) The WT, *ΔCgMsb2*, and *ΔCgMsb2Sho1* strains were cultured on PDA medium in plates at 25 °C for 5 days. (**D**) Conidial production on PDA medium in plates after 5 days. Error bars represent the standard deviation for three independent replicates. Asterisks indicate significant differences (** *p <* 0.01). (**E**) Colony diameter of the strains cultured on PDA medium in plates.

There were no significant differences in the length/width ratios of the wild-type (WT), *ΔCgMsb2*, and *ΔCgMsb2Sho1* conidia (Figure 1B). Interestingly, the spore yield was much higher for *ΔCgMsb2Sho1* than for the WT control (Figure 1A,D). Regarding growth, we measured the diameters of the WT, *ΔCgMsb2*, and *ΔCgMsb2Sho1* colonies starting from day 2 of the growth period (Figure 1E). The colony growth rate was slightly higher for the WT control (12.48 mm/day) than for the *ΔCgMsb2* (11.37 mm/day) and *ΔCgMsb2Sho1* (11.76 mm/day) mutants.

### 3.2. RNA Sequencing (RNA-seq) Profiles and Identification of Differentially Expressed Genes (DEGs)

To explore the transcriptomes of *C. gloeosporioides* associated with Msb2 and Sho1, biological replicates of the control samples (T0-1, T0-2, and T0-3) and the mutant samples (M0-1, M0-2, and M0-3 as well as MS0-1, MS0-2, and MS0-3) were included in the transcriptome analysis. The Q scores exceeded 30 for >85% of the reads in all nine libraries (Table 1), reflecting the high quality of the RNA-seq data. More than 1.21 Gb of clean bases were obtained per library.

**Figure 2:**
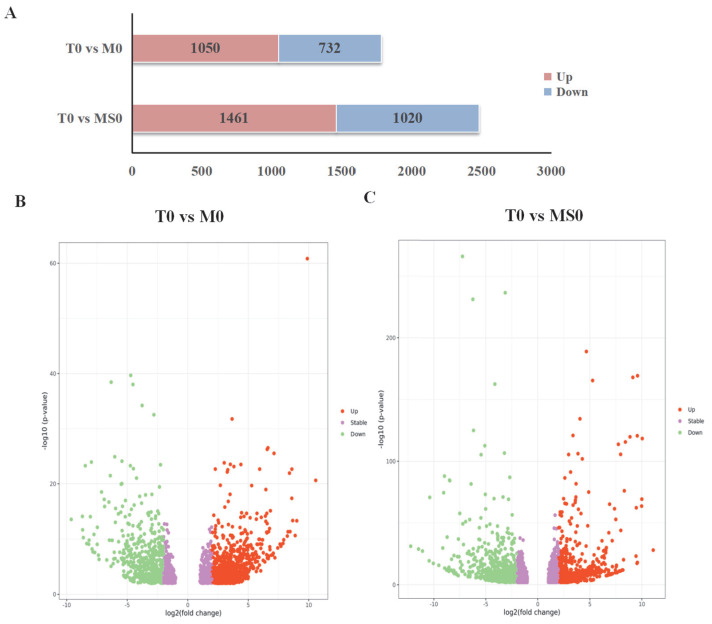
Differentially expressed genes (DEGs) in the *ΔCgMsb2* and *ΔCgMsb2Sho1* mutant strains. (**A**) Number of DEGs in the *ΔCgMsb2* and *ΔCgMsb2Sho1* strains. Red and blue indicate upregulated and downregulated expression, respectively. (**B**,**C**): Volcano plots presenting the distribution of DEGs (fold-change ≥ 2 and adjusted *p <* 0.01) in the *ΔCgMsb2* mutant (compared with the control) (**B**) and in the *ΔCgMsb2Sho1* mutant (compared with the control) (**C**). Red and green indicate significantly upregulated and downregulated DEGs, respectively, whereas purple indicates DEGs that are not significant.

Analyzing the DEGs among the control and mutant samples may be useful for further clarifying the regulatory effects of the signal-transduction-related *C. gloeosporioides* membrane proteins. Genome-wide transcriptional profiling of M0 and MS0 detected substantial transcriptional changes to 19–26% of the genes. A total of 1788 DEGs were detected in the T0 vs. M0 comparison, of which 1056 and 732 were upregulated and downregulated, respectively. Additionally, 2481 DEGs were detected in the T0 vs. MS0 comparison, of which 1461 and 1020 were upregulated and downregulated, respectively. The overall DEG distribution is presented in Figure 2A. The significant DEGs (i.e., adjusted fold-change ≥ 2 and corrected *p <* 0.01) (Figure 2B,C).

### 3.3. DEGs among the Transcriptomes

**Figure 3:**
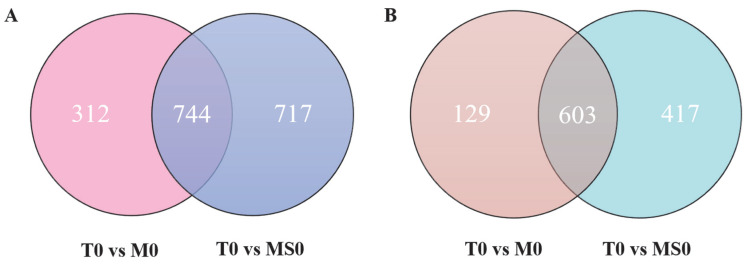
(**A**) Venn diagram presenting the number of upregulated DEGs in the *ΔCgMsb2* and *ΔCgMsb2Sho1* mutants. The numbers in each circle represent the number of DEGs between two samples. The overlapping parts of the circle represent commonly expressed DEGs in the two samples. (**B**) Venn diagram presenting the number of downregulated DEGs in the mutant samples. The numbers in each circle represent the number of DEGs between two samples. The overlapping parts of the circle represent commonly expressed DEGs in the two samples.

To explore gene expression similarities and differences among transcriptomes, we generated Venn diagrams to profile the DEG distribution between the T0 vs. M0 and T0 vs. MS0 comparisons. Of the upregulated DEGs, 312 were specific to the T0 vs. M0 comparison, whereas 717 were specific to the T0 vs. MS0 comparison. However, 744 upregulated DEGs were common to both comparisons (Figure 3A). There were slightly fewer downregulated DEGs in the T0 vs. M0 comparison than in the T0 vs. MS0 comparison (Figure 3B). The Gene Ontology (GO)-based functional annotations indicated that molecular-function-related GO terms were significantly enriched among ten upregulated DEGs (T0 vs. M0 and T0 vs. MS0), of which five were associated with transmembrane transport. Interestingly, three of these five DEGs were identified as genes encoding ATP-binding cassette (ABC) transporters (i.e., BEA3 and FUM19) (Table 2). Moreover, half of the 10 most downregulated DEGs (T0 vs. M0 and T0 vs. MS0) were identified as hydrolase-related genes (Table 2). The downregulation of glycosyl hydrolase in *C. gloeosporioides* affects the penetration of plant epidermal barrier.

### 3.4. GO Analysis of DEGs

**Figure 4:**
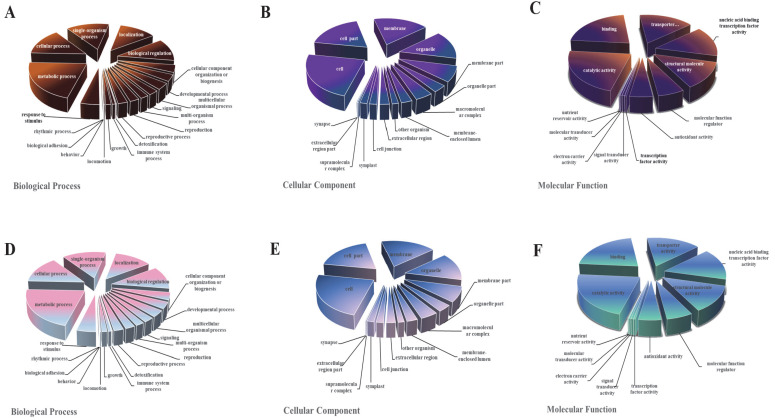
The Gene Ontology (GO) classification of DEGs. GO classification of identified DEGs based on their functional annotations in response to various samples was conducted according to the GO resource (http://geneontology.org/, accessed on 17 January 2022). (**A**–**C**) Indicate biological processes, cellular components, and molecular function under mutant CgMsb2, while (**D**–**F**) indicate biological processes, cellular components, and molecular function under mutant CgMsb2sho1, respectively.

All DEGs were functionally characterized and classified according to a GO term enrichment analysis. The DEGs were distributed in the three main GO categories (i.e., biological process, cellular component, and molecular function). In the M0 samples, metabolic process (GO: 0008152), cell (GO: 0005623), and catalytic activity (GO: 0003824) were the main GO terms assigned to the DEGs in the molecular function, cellular component, and biological process categories, respectively (Figure 4). The MS0 samples lacking Msb2 and Sho1 had similar GO classifications and ratios. The DEGs of metabolic process obviously changed, which indicates that CgMsb2 and CgSho1 affect the process of material and energy exchange.

### 3.5. Kyoto Encyclopedia of Genes and Genomes (KEGG) Pathway Enrichment Analysis of DEGs

**Figure 5:**
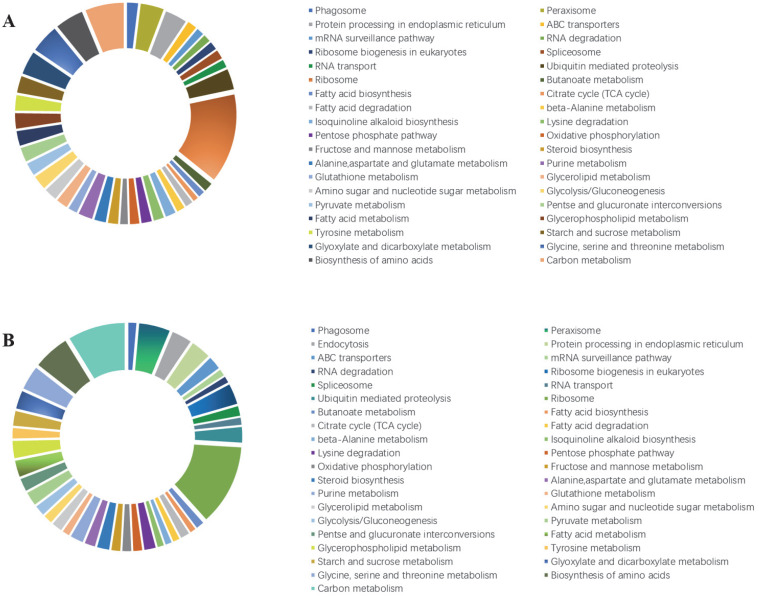
Kyoto Encyclopedia of Genes and Genomes (KEGG) pathway enrichment analysis of the DEGs in the *Colletotrichum gloeosporioides* mutants. (**A**) T0 vs. M0 comparison. (**B**) T0 vs. MS0 comparison. The KEGG pathways were classified into hierarchical categories according to the KEGG website (https://www.kegg.jp/kegg/kegg2.html, accessed on 17 January 2022). T0, wild-type *C*. *gloeosporioides* conidia; M0, *ΔCgMsb2* conidia; MS0, *ΔCgMsb2Sho1* conidia.

The distribution and ratio of enriched KEGG pathways among the identified DEGs are presented in Figure 5. Ribosome, carbon metabolism, biosynthesis of amino acids, and amino acid metabolism (glycine, serine, and threonine) were among the top five enriched KEGG pathways in both mutants. We mapped the enriched KEGG pathway network for the DEGs (Supplementary Figure S1).

### 3.6. Hydrolase-Related DEGs

**Figure 6:**
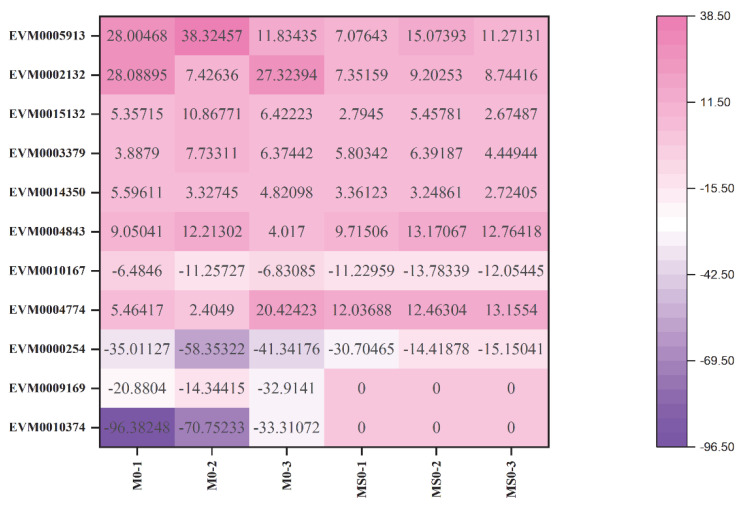
Heat map of glycosyl-hydrolase-related differentially expressed genes in the T0 vs. M0 and T0 vs. MS0 comparisons. Pink and purple indicate relatively high and low expression levels, respectively.

Compared with the corresponding expression levels in the T0 transcriptome, 52 genes encoding 22 glycosyl hydrolase family members had upregulated or downregulated expression levels in the mutant transcriptomes (Supplementary Table S1). The gene encoding glycosyl hydrolase family 31 (*GH31*; EVM0015745) was the most differentially expressed (i.e., upregulated nearly 204 times). This enzyme is an alpha-glucosidase that is indispensable for glucose metabolism. Additionally, many of the genes were identified as β-glucosidase genes belonging to the cellulase group, including *GH3* (EVM0005913, EVM0002132, EVM0015132, EVM0003379, and EVM0014350), *GH5* (EVM0004843), and *GH17* (EVM0010167 and EVM0004774). Of these β-glucosidase genes, the downregulated expression of only *GH17* (EVM0010167) was verified by quantitative real-time PCR (qRT-PCR). In *C. gloeosporioides*, *GH17* (EVM0010167) may contribute to plant cell wall degradation during the infection of host plants. In addition to the above-mentioned β-glucosidases, *GH7* and *GH12* were annotated as glycoside hydrolases related to cellulose decomposition. Notably, only one of *GH18* (EVM0009169) and *GH25* (EVM0010374) were revealed to be related to chitin hydrolases. The *GH25* and *GH18* genes were detected in the *ΔCgMsb2*, but not in the *ΔCgMsb2Sho1*, suggesting they are closely associated with CgMsb2 (Figure 6).

### 3.7. Differential Expression of Candidate ABC Transporter Genes

The ABC proteins, which are present in all living cells, are important because they are often associated with the multidrug resistance of microbial pathogens [32]. An examination of gene expression patterns in the *ΔCgMsb2* and *ΔCgMsb2Sho1* mutants detected 12 ABC-transporter-encoding DEGs (fold-change ≥ 2 and corrected *p* < 0.01). The expression levels of most of the ABC transporter genes were significantly upregulated during appressorium development, especially *BEA3* (EVM0009311) (i.e., upregulated nearly 18 times). This gene belongs to the same family as the gene encoding ABC transporter B family member 4 (ABC4), which is reportedly involved in appressorium formation and is a pathogenicity factor in *Magnaporthe* species [33]. Moreover, the expression levels of *Pmd1* and *FUM19*, which encode ABC transporters, were, respectively, 12.48 times and 9.17 times higher in the M0 samples than in the T0 samples (Table 2). Significant changes with ABC transporters may affect the transport of *C. gloeosporioides* nutrients and lipids, as well as the transport of secondary metabolites

### 3.8. DEGs Involved in the MAPK Signaling Pathway

The MAPK signaling pathway is necessary for the infection of susceptible hosts by plant pathogens [34]. Regarding *C. gloeosporioides*, MAPK signaling pathway components are crucial for appressorium formation. The upregulated expression of *PPS1* (EVM0006436) was revealed by the T0 vs. M0 and T0 vs. MS0 comparisons. This upregulated expression likely inactivated MAPK via the negative regulation of protein phosphorylation, thereby preventing the normal transmission of the MAPK signaling pathway. Conversely, the expression of genes related to guanine-nucleoside-binding protein subunit β (EVM0000353) was downregulated in the M0 and MS0 samples by 7.13 times and 8.90 times, respectively. The EVM0000353 gene influences the MAPK scaffold activity and positively regulates protein phosphorylation. The detected downregulated expression of the EVM0000353-related genes in the mutants may adversely affect the normal formation of appressoria after spores germinate by disrupting the MAPK signaling pathway.

### 3.9. Verification of Gene Expression by qRT-PCR

**Figure 7:**
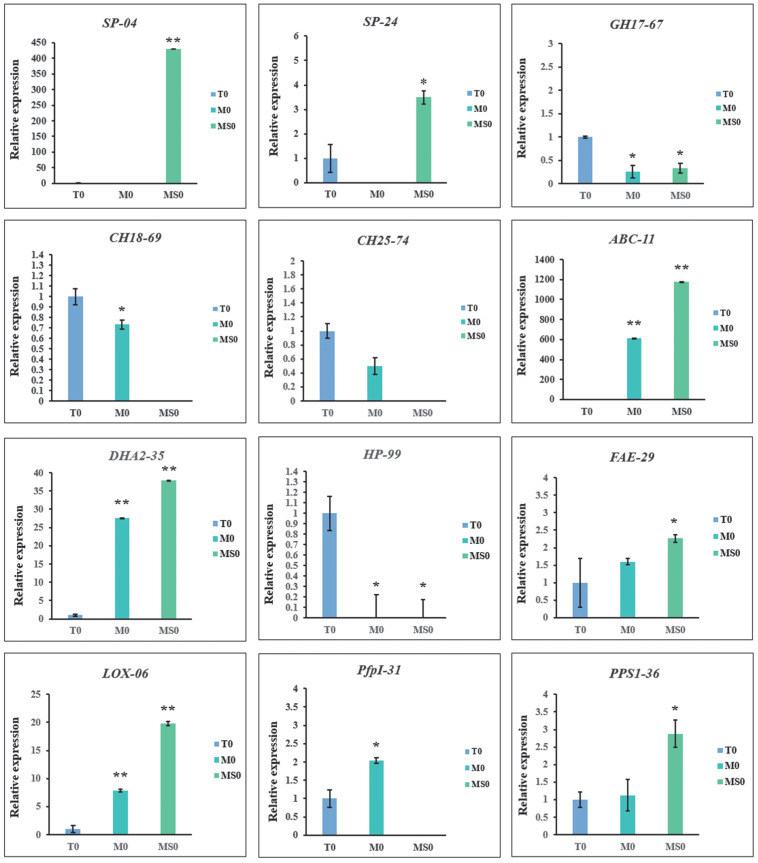
Results of the qRT-PCR validation of key candidate DEGs in the M0 and MS0 samples. Error bars represent the standard deviation for three independent replicates. Asterisks indicate significant differences (** *p* < 0.01; * *p* < 0.05).

To verify the accuracy of the transcriptomic data, we selected specific DEGs in the M0 and MS0 samples encoding glycosyl hydrolases, MAPKs, and proteins related to sporulation and transport for a qRT-PCR analysis. For the 12 selected DEGs (Supplementary Table S2), the qRT-PCR data were consistent with the RNA-seq data (Figure 7), implying the RNA-seq results were reliable.

We also performed a qRT-PCR analysis to verify the RNA-seq data for the upregulated genes (EVM0005624 and EVM0009704) related to conidia production. Both of these genes were not detected in the M0 samples, suggesting they were specifically expressed in the MS0 samples, which affected spore production to some extent.

## 4. Discussion

In this study, 19–26% of the *C. gloeosporioides* genes were differentially regulated in the *ΔCgMsb2* and *ΔCgMsb2Sho1* mutants. To elucidate the regulatory mechanisms of the membrane proteins Msb2 and Sho1 in plant pathogens, a few transcriptomic studies on basidiomycetous fungi have been conducted. In *U. maydis*, Sho1 and Msb2 are two membrane receptor proteins that regulate the expression of genes encoding plant cell-wall-degrading enzymes, which is consistent with our findings regarding some cellulose-degrading enzymes and pectin lyases. However, we specifically screened for spore-producing genes related to phenotypic changes, MAPKs, and ABC transporters. On the basis of the results presented herein, the mutations in *ΔCgMsb2* and *ΔCgMsb2Sho1* triggered major changes in gene transcription. In this study, we revealed gene expression differences between *ΔCgMsb2* and *ΔCgMsb2Sho1*. The RNA-seq analyses suggested that the mutations in *ΔCgMsb2* and *ΔCgMsb2Sho1* considerably affected catalytic activities, transporter activities, and oxidoreductase activities. However, the expression analyses indicated that the extent of the changes to gene expression was generally greater in *ΔCgMsb2Sho1* than in *ΔCgMsb2*, which is in accordance with our observation that the pathogenicity of *ΔCgMsb2Sho1* was significantly more adversely affected than that of *ΔCgMsb2*.

Fungal cell walls are mainly composed of carbohydrate polymers and glycoproteins. The conserved carbohydrate components within fungal cell walls include β-glucans, chitin, and mannans [35]. Structurally, chitin, β-glucans, and mannans tend to be in the inner, middle, and outer parts of the cell wall, respectively. Hence, mannans cover the β-glucans and chitin, which impairs fungal recognition. Glycosyl hydrolase is a critical enzyme for the decomposition of the above-mentioned polysaccharides. Many glycosyl hydrolases that hydrolyze diverse substrates were derived from the same ancestor and are structurally and functionally similar. Among the DEGs identified in this study, the glycosyl hydrolase family 18 genes are mainly involved in the amino sugar and nucleotide sugar metabolic pathway. More specifically, *GH18*-related enzymes convert chitin to chitobiose or N-acetylglucosamine. Additionally, the glucan endo-1,3-beta-glucosidase eglC in *GH17* contributes to the conversion of beta-1,3-glucan to glucose during starch and sucrose metabolism. The cell surface mannoprotein MP65 of *GH17* is one of the main components of the cell wall of fungus. Mannoproteins are formed through the glycosylation of specific proteins. The initial glycosylation generally occurs in the endoplasmic reticulum, after which the protein is further processed in the Golgi body [36]. Mannosylation patterns may vary broadly among fungal species. Moreover, heterogeneous mannosylation reactions may occur in the strains and morphological types of a single species [37]. We believe that the cell surface mannoprotein MP65 in *C. gloeosporioides* is the result of a long-term evolution of a specific glycosyl hydrolase.

When plant pathogens sense the plant cell wall composed of cellulose, hemicellulose, and polysaccharide components [38], they secrete a variety of glycosyl hydrolases to enable the infection of susceptible hosts. These enzymes convert the plant polysaccharides to oligosaccharides or monosaccharides, while also destroying the cell wall structure to facilitate the penetration of the epidermal barrier [39]. The secretion of cell-wall-degrading enzymes is an important process during the infection of plants by pathogens [40]. The glycosyl hydrolases secreted by pathogenic fungi are closely associated with infection characteristics. In an earlier study on *Magnaporthe grisea*, an RNA interference-based strategy was applied to decrease the expression of *GH6* and *GH7* family genes [41], which was detrimental to cell wall degradation and led to a significant decrease in pathogen virulence. When *C. gloeosporioides* changes from a biotroph to a necrotroph, glycosyl hydrolase genes are highly expressed and there is a significant increase in virulence [42]. However, to increase the degradation of cellulose, lignocellulose should be converted to oligosaccharides or monosaccharides. We identified a lytic polysaccharide mono-oxygenase, which can effectively degrade the cellulose in plant cell walls.

After glycosylases hydrolyze various sugar-containing compounds to generate monosaccharides, ABC transporters bind ATP and use the energy to drive the transport of various molecules. A previous study demonstrated that ATP serves as hydrolytic energy to drive cellular activities, including the input and export of nutrients and lipids, respectively [43]. Fungal transporter proteins have a wide range of biological functions. They are crucial for the pathogenicity of plant pathogenic fungi [44,45]. The prototypical ABC protein comprises two transmembrane domains and two nucleotide-binding domains [32]. The ABC protein family has been divided into nine subfamilies (A–I) according to the nucleotide-binding domain structure and amino acid sequence [46]. In *C. gloeosporioides*, an ABC protein (CgABCF2) is reportedly required for appressorium formation and plant infections [47]. In *Fusarium graminearum*, FgABC1 is involved in the secretion of fungal secondary metabolites [48]. We detected the upregulated expression of several ABC transporter genes (EVM0009311, EVM0003035, EVM0000077, EVM0009520, EVM0012444, EVM0001131, and EVM0010668) in the T0 vs. M0 and T0 vs. MS0 comparisons. We speculate that the upregulated genes are related to the transmembrane transport of fungal secondary metabolites. Pathogens produce various secondary metabolites, such as quinones, flavonoids, and phenols, to protect against adverse environmental conditions. Additionally, ABC transporters play an important role in the accumulation and excretion of compounds. Some ABC proteins (e.g., ABCB1) function as transporters and channel proteins that help maintain intracellular homeostasis. The upregulated expression of ABC transporter genes in this study may be relevant for future research on pathogen secondary metabolism and the regulation of the related genes in *ΔCgMsb2* and *ΔCgMsb2Sho1*.

An earlier investigation determined that a MAPK cascade controls the infection-related morphogenesis of *C. gloeosporioides* [49]. In *Botrytis cinerea*, Msb2 regulates host surface sensing and penetration via the BMP1–MAPK signaling pathway [50]. The upregulated protein phosphatase gene *PPS1* (EVM0006436) is a MAPK phosphatase (MKP) that functions as an important negative regulator of MAPK activity. More specifically, MKP inactivates MAPK by dephosphorylating the enzyme. There are three important members of the MAPK family, namely ERK, stress-activated protein kinase (JNK or SAPK), and p38, which can be regulated by MKP. Interestingly, PPS1 inhibits the formation of ERK as well as the activation of downstream transcription factors via dephosphorylation.

In this study, the *ΔCgMsb2* and *ΔCgMsb2Sho1* mutants differed from the WT control in terms of gene expression. Thus, we speculated that the downregulated expression of some genes in *ΔCgMsb2* and *ΔCgMsb2Sho1* during the growth stage (relative to the corresponding expression in the initial spore stage) is conducive to the complete induction of the transcriptional network in the developing fungus. Additionally, the Src homology 3 (SH3) domain is a conserved small module in the membrane protein Sho1. The presence of the SH3 domain enables proteins to interact with specific proline-rich sequences in protein partners [51]. The SH3 domain mediates interactions associated with the regulation of signal transduction events. Therefore, the SH3 domain of CgSho1 likely contributes to the synergistic effects of CgSho1 and CgMsb2. The combined activities of CgSho1 and CgMsb2 have a greater effect on gene expression than the CgMsb2 activity alone.

According to our results, the absence of CgMsb2 and CgSho1 in *C. gloeosporioides* has inhibitory effects on some MAPK pathways (e.g., ERK and JNK). The qRT-PCR data generated in this study verified some of these changes, while also providing evidence of the importance of hydrolases in carbohydrate metabolism. Additionally, DEGs associated with ABC transporter upregulated in *ΔCgMsb2* and *ΔCgMsb2Sho1* in response to the transport of carbohydrate and secondary metabolites, and therefore ABC transporters apparently play an important role in metabolic processes. The study findings revealed that CgMsb2 and CgSho1 direct central transcription networks that enable *C. gloeosporioides* to penetrate the plant hosts. It is clear that Msb2 and Sho1 trigger major differences in *C. gloeosporioides*. Furthermore, the results described herein may provide insights into the effects of the membrane proteins Msb2 and Sho1 on the transcriptional networks associated with other plant diseases caused by fungi. This study should lay an important foundation for the further study and verification of related gene functions in MAPK pathway, glycosyl hydrolase and ABC transporters.

## Figures and Tables

**Table 1:** RNA sequencing data for the T0, M0, and MS0 samples. Note: T0, wild-type *C*. *gloeosporioides* conidia; M0, *ΔCgMsb2* conidia; MS0, *ΔCgMsb2Sho1* conidia.

Sample	Data (bp)	Clean Reads	Q30 (%)	Total Mapping (%)	Uniquely Mapping (%)
T0	6,286,653,483	21,019,807	94.81%	91.01%	90.66%
M0	8,905,212,135	29,816,717	94.01%	95.79%	95.54%
MS0	9,194,480,861	30,799,568	94.74%	95.99%	95.74%

**Table 2:** Upregulated and downregulated genes in the *ΔCgMsb2* and *ΔCgMsb2Sho1* mutants.

Condition	ID	Fold Change	*p* Value	Annotation
T0-M0-up	EVM0005631	807.4227	1.46 × 10^−^^61^	Glyoxylate reductase
	EVM0008231	277.6544	0.0000608	DJ-1/PfpI family, transcription factor
	EVM0015745	204.4752	1.14 × 10^−10^	Glycosyl hydrolase family 31
	EVM0012935	133.5726	2.74 × 10^−9^	MFS transporter
	EVM0008406	106.5425	3.04 × 10^−27^	Linoleate 9S-lipoxygenase 1
	EVM0009311	18.0056	0.001193	BEA3, ABC transporter
	EVM0003035	12.4763	0.000566	pmd1, ABC transporter
	EVM0006436	11.2868	0.000732	PPS1, Protein phosphatase
	EVM0000077	9.1756	0.002521	FUM19, ABC transporter
	EVM0007629	8.7358	5.10 × 10^−14^	Feruloyl esterase
T0-M0-down	EVM0008257	316.9458	3.09 × 10^−19^	Pectate trisaccharide-lyase
	EVM0002108	62.7619	1.17 × 10^−20^	Pectate lyase
	EVM0010374	55.0161	7.21 × 10^−9^	Polysaccharide deacetylase
	EVM0010120	50.3543	1.40 × 10^−6^	Glycosyl hydrolase family 12
	EVM0006919	31.4844	0.000891085	erg-4, methyltransferase
	EVM0009169	15.2328	0.001032984	Glycosyl hydrolase family 18
	EVM0013863	14.7652	1.39 × 10^−6^	Cutinase
	EVM0006990	13.1765	9.29 × 10^−10^	Scytalone dehydratase
	EVM0010167	7.7034	1.84 × 10^−5^	Glycosyl hydrolase family 17
	EVM0000353	7.1256	7.81 × 10^−8^	Guanine nucleotide-binding protein
T0-MS0-up	EVM0002782	680.7201	5.15 × 10^−63^	Carboxylesterase
	EVM0005631	577.5573	4.67 × 10^−170^	Glyoxylate reductase
	EVM0005555	156.8511	2.32 × 10^−12^	Nitrogen assimilation transcription factor
	EVM0015745	90.8438	4.78 × 10^−14^	Glycosyl hydrolase family 31
	EVM0009311	19.3072	3.04 × 10^−10^	BEA3, ABC transporter
	EVM0008406	14.4585	3.47 × 10^−34^	Linoleate 9S-lipoxygenase 1
	EVM0006436	4.8744	0.00386738	PPS1, protein phosphatase
	EVM0000077	7.3816	0.000153315	FUM19, ABC transporter
	EVM0005624	4.9106	7.34 × 10^−3^	E3 ubiquitin-protein ligase, sporulation
	EVM0009704	4.1047	5.11 × 10^−5^	conidium formation
T0-MS0-down	EVM0001785	834.5262	1.79 × 10^−10^	Pectate lyase
	EVM0002699	588.0388	5.66 × 10^−85^	Hypothetical protein
	EVM0008890	321.3349	3.59 × 10^−33^	YheN, polysaccharide deacetylase
	EVM0002162	275.5874	1.33 × 10^−37^	isp4, transporter protein
	EVM0006990	70.4276	1.47 × 10^−15^	Scytalone dehydratase
	EVM0006183	15.7665	1.67 × 10^−18^	cAMP-dependent protein kinase
	EVM0010120	13.0258	8.16 × 10^−9^	Glycosyl hydrolase family 12
	EVM0000353	8.8958	2.96 × 10^−25^	Guanine nucleotide-binding protein
	EVM0013863	8.0850	0.000000818	Cutinase
	EVM0001921	6.6476	0.005086478	Inositol 5-phosphatase
	EVM0010374	4.8228	0.007172842	Polysaccharide deacetylase

## Data Availability

Data is contained within the article or Supplementary Material.

## Supplementary Materials

The following supporting information can be downloaded at: https://www.mdpi.com/article/10.3390/jof8020207/s1, Figure S1. The enrichment network of differentially expressed gene KEGG; Table S1: DEGs involved in glycosyl hydrolase; Table S2: Candidate genes for primer pairs; Table S3. Primer sequence used for qRT-PCR in this study.
