# Supplementary material for: Transcriptional Network in Colletotrichum gloeosporioides Mutants Lacking Msb2 or Msb2 and Sho1
Source: J Fungi (Basel). 2022 Feb 21;8(2):207. doi: 10.3390/jof8020207 (PMC8878819; doi:10.3390/jof8020207)
Supplement: Supplementary file 1 [file jof-08-00207-s001.zip › jof-1581818-supplementary.pdf]

Table S1. DEGs involved in glycosyl hydrolase

GHs	ID	Fold Change （M0）	p-Value (M0)	Fold Change （MS0）	p-Value (MS0)
15	EVM0005982	5.9219	0.000135	2.969938	6.92E-07
5	EVM0004843	8.4268	4.50E-06	11.8833	9.04E-62
47	EVM0006394	6.6360	3.13E-07	5.380609	0.000123
88	EVM0009009	8.5642	0.001586	7.31227	2.04E-11
92	EVM0010947	11.7875	1.43E-07	6.002521	3.85E-06
38	EVM0012553	9.2542	0.000954	5.614916	0.000395
18	EVM0009169	-15.2329	0.001033	/	/
25	EVM0010374	-55.0161	7.21E-09		
79	EVM0000254	-42.9259	5.26E-24	-17.8649	3.58E-09
2	EVM0002091	5.9521	0.002614	/	/
3	EVM0002132	20.9464	1.51E-05	8.432759	2.31E-09
	EVM0003379	5.9985	3.58E-05	5.548246	9.51E-15
	EVM0005913	52.1081	4.73E-07	2.28111	4.65E-21
	EVM0014350	4.5815	0.000471	3.111299	5.87E-08
	EVM0015132	7.5490	4.28E-07	3.642396	1.25E-09
7	EVM0003261	-99.1316	0.000617	-66.1625	1.29E-05
	EVM0005737	9.4482	5.48E-09	/	/
	EVM0010243	8.6154	7.40E-08	15.31504	1.16E-08
10	EVM0001249	-10.3215	8.37E-19	-12.1762	3.58E-09
	EVM0005978	-49.2774	2.99E-07	-20.0943	0.000858
	EVM0015905	-10.2461	9.32E-06	-4.4284	9.92E-12
12	EVM0001779	-5.3151	0.001132	-4.65595	1.26E-07
	EVM0009853	-11.3241	7.17E-07	-5.51651	4.14E-10
	EVM0010120	-50.3543	1.40E-06	-13.0258	5.65E-09
	EVM0015211	5.2908	0.004284	/	/
16	EVM0001285	-5.5020	7.87E-06	-8.16779	1.94E-06
	EVM0005991	-8.8404	3.00E-33	-11.6635	3.55E-11
	EVM0006710	-18.7786	6.28E-35	-6.87329	3.78E-11
	EVM0010284	2.1228	0.001008	1.990919	2.66E-10
	EVM0015862	-50.0492	1.45E-15	-76.4086	8.40E-10
	EVM0016050	-20.4645	4.36E-08	-7.22666	9.08E-06
17	EVM0004774	9.4311	0.001493	12.55144	1.04E-34
	EVM0010167	-7.7034	1.84E-05	-12.267	9.09E-07
28	EVM0001425	-32.2580	1.10E-08	-76.8918	1.40E-20
	EVM0015377	-73.5372	0.001226	-177.412	0.005687
	EVM0015584	-15.9953	6.57E-05	-17.926	0.000953

	EVM0016027	24.7641	4.04E-06	8.864885	1.30E-09
31	EVM0000329	3.4876	0.007654	/	/
	EVM0002802	4.6349	0.004342	/	/
	EVM0015745	204.4751	1.14E-10	90.84385	4.78E-14
43	EVM0003464	4.6162	0.00216	/	/
	EVM0006952	6.3349	0.000254	7.609333	2.68E-05
61	EVM0000498	8.5701	0.001709	/	/
	EVM0001634	-14.4325	9.67E-06	-17.0186	1.30E-18
	EVM0005936	32.8636	3.29E-07	30.23453	3.90E-12
	EVM0007129	-6.8974	0.000195	-15.278	1.03E-34
	EVM0009521	-32.1924	2.86E-05	-29.4654	5.78E-23
	EVM0011109	-15.3415	1.19E-05	-22.4463	8.16E-09
	EVM0011511	83.7432	2.33E-15	22.88058	2.55E-10
	EVM0013798	15.6812	1.01E-06	21.00574	3.80E-14
76	EVM0009284	-8.4399	0.008009	-4.25996	6.42E-50
	EVM0012154	-6.8544	0.000304	/	/

Table S2. Candidate genes for primer pairs

ID	Fold Change （M0）	p-Value (M0)	Fold Change （MS0）	p-Value (MS0)	Annotation
EVM0009704	/	/	4.1047	5.11E-05	sporulation
EVM0005624	/	/	4.9106	0.007336	sporulation
EVM0010167	-7.7034	1.84E-05	-12.2670	1.3E-18	glycosyl hydrolase 17
EVM0009169	-15.2328	0.001032984	/	/	glycosyl hydrolase 18
EVM0010374	-55.0161	7.21E-09	/	/	glycosyl hydrolase 25
EVM0009311	18.0056	0.001193	19.3072	3.04E-10	ABC transporter
EVM0012935	133.5726	2.74E-09	187.7427	2.52E-106	DHA2 family
EVM0002699	-72.7191	1.21E-16	588.0388	5.66E-85	hypothetical protein
EVM0008406	106.5425	3.04E-27	14.4585	3.47E-34	Linoleate9S-lipoxygenase1
EVM0007629	9.87244	1.26E-18	27.5888	1.15E-28	Feruloyl esterase
EVM0008231	277.6544	6.08E-05	/	/	DJ-1/PfpI family
EVM0006436	11.2868	0.000732	4.8745	0.0038673	PPS1, Protein phosphatase

Table S3. Primer sequence used for qRT-PCR in this study.

SP04 forward primer	AAGCACGCCTCTGTCATGAA
SP04 reverse primer	GCGTCCTTAACATCGAGCTT
SP24forward prime	GCAGGCAAGGAGTTCGATG
SP24 reverse primer	GTGTGTATGTGCGTGGAAGG
GH17-67forward prime	ACATCCGGAACAGCATCGA
GH17-67 reverse primer	CTCTCCGACACACTCTCGTC
GH18-69forward prime	CAACCAGCCCCAAGATGTTG
GH18-69 reverse primer	TCCAATTCCATCCCCAACCA
GH2574forward prime	CGGATGGCTGGAGAATGATAT
GH2574 reverse primer	GCTTCTTCCAACACAGCCTC
ABC-11forward prime	TCGCAAAGCTTGGAGGAGAT
ABC-11 reverse primer	GCTGTTGGTGCTGATATCCA
DHA2-35 forward prime	ACAAGATGTTCGTCAAGATGCA
DHA2-35 reverse primer	TCTCATCAGCATTGTCCTCCT
HP-99forward prime	TTTGATGAAGCACTCGGGTC
HP-99 reverse primer	TCAACCTCCTCAACGAAGCT
FAE29forward prime	ATGGGCAATGTCAGGCTGA
FAE29 reverse primer	TCTCTTTAAGAACCACGGATCC
LOX-06 forward prime	CTCGTCGAGGGCATGTCC
LOX-06 reverse primer	CTTCCACCCGACGACCTC
PfpI-31 forward prime	TGGCGCGATTGATACTCCAA
PfpI-31 reverse primer	ATCAACACTCCAGGCGGC
PPS1-36forward prime	CCCATCTGGTACTACTGCGC
PPS1-36 reverse primer	GCTTAACCGGCGGAATCAC

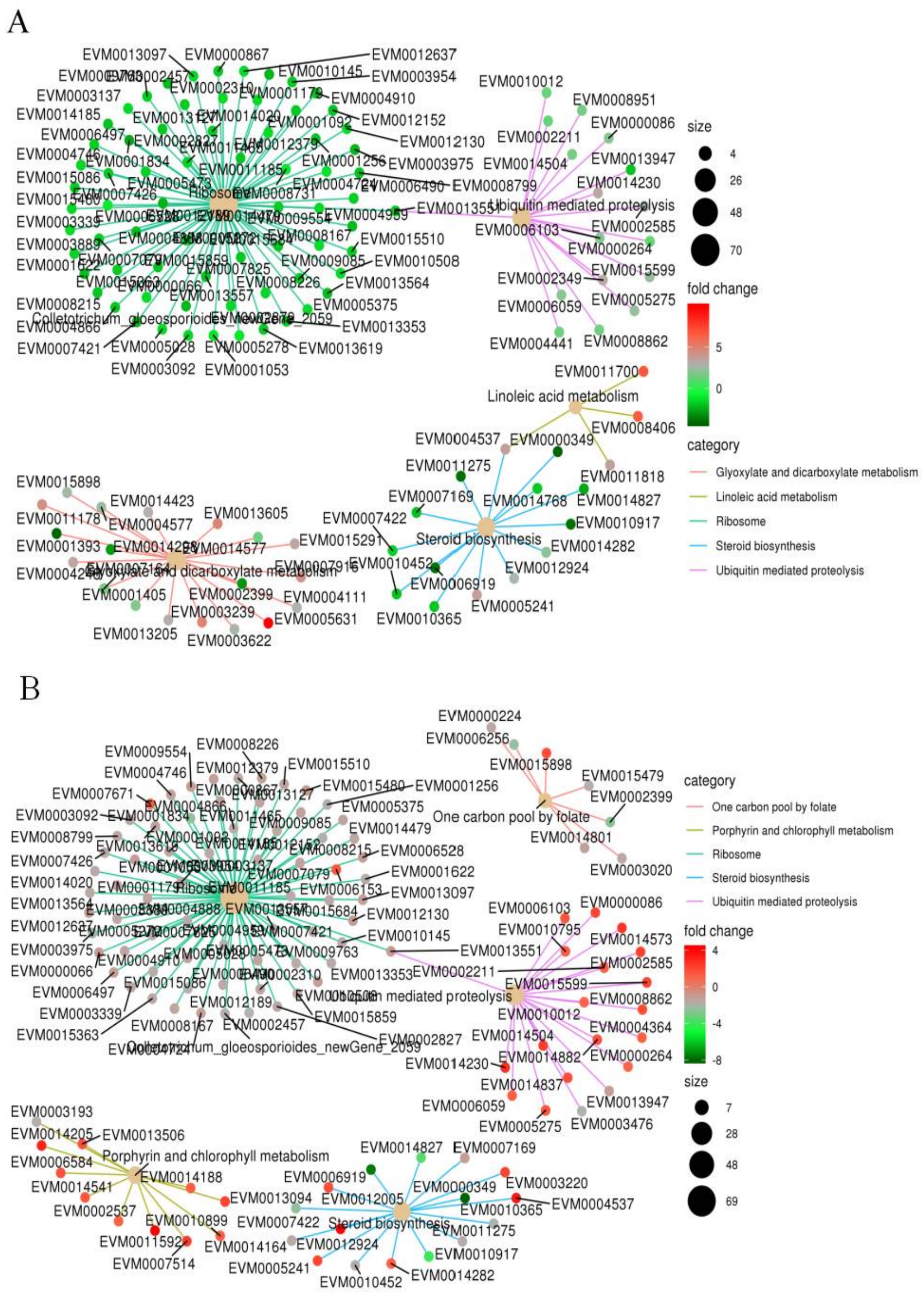

Figure S1. The enrichment network of differentially expressed gene KEGG. (A) means T0 versus M0 and (B) meams T0 versus MS0. The red dots represent up-regulated genes, while the green dots represent down-regulated genes.
